# Breaking Paradigms: Students in Perspective

**DOI:** 10.6061/clinics/2018/e434

**Published:** 2018-09-24

**Authors:** Gustavo Rosa Gameiro, Henrique Branzani da Silva

**Affiliations:** Graduation in Medicine, Faculty of Medicine FMUSP, University of Sao Paulo, Sao Paulo, SP, BR

On Wednesday at 2:30 pm, after attending all of my morning classes and having lunch, I intended to rest. However, on my way home, as I climbed the stairs of the college, I ran into the dean, Professor Auler, in a lab coat, who was coming down the stairs and heading for the intensive care unit (ICU) to visit some patients. To my total surprise, he invited me to follow him.

In a few minutes, I found myself in a circle of approximately 10 professionals, including doctors, nurses and physiotherapists, discussing the bedside management of each patient, including enigmatic cases and apparently less complex ones. The discussion was filled with acronyms, ideas and citations of studies recently published in respected scientific journals.

As I contemplated so many professionals working for the health of one individual, I was beginning to grasp a true dance of knowledge; I felt a little pang of angst, for it looked as if I added very little to the discussion. It seemed to me that as a student, I was only disturbing them with my questions and doubts. At the end of the visit that day, I took home not only the new knowledge and some doubts but also a certain feeling of discomfort: what is the role of students in health services?

During a student's very first contact with the hospital at the basic health unit or the outpatient clinic, we realize how asymmetric our relationship with these institutions will be. On one hand, there are freshmen who are unable to recognize an obvious heart murmur or even a classic sign of massive ascites. On the other hand, hospitals are large organizations with hundreds of patients, multidisciplinary teams and an infrastructure that sometimes includes a kitchen, pharmacy, laundry facility, waste collection center and more.

At first, it is difficult to find our place, as we do not have the experience to discuss medical conduct based on the latest update published in a reputed journal. We have difficulty talking to patients about their illnesses, it takes us longer to carry out anamnesis and physical examinations, and our medical visits are longer due to elementary questions.

During one of my weekend rotations in the infectious disease ward, for example, I was supposed to see patients quickly and discuss them briefly with the attending physician. Something that was supposed to take a few hours ended up stretching for a whole day, since the attending physician was interested in answering all my questions. It was the longest visit I ever attended and by far the most didactic one.

Contrary to what one may expect, although students need more time to talk and perform physical examinations, patients do not seem to think that longer examinations are boring or tiresome; they actually recognize how humane and intense a longer consultation may be, which is something that could go unnoticed in a quicker anamnesis but is more likely to be noticed by a student.

The health system continues to function despite the presence of the student, and I have already caught myself wondering if it would be more efficient if we were not there.

It is undoubtedly inside the various health facilities where we grow academically: we begin to understand the main syndromes, we learn to treat the most common clinical conditions and we learn how to behave in emergency situations. We also understand that the hierarchy present in the health system is not as simple as it seems. Personally, we build relationships with patients and their families and with our friends, coworkers, mentors and superiors. Emotionally, we experience human suffering so closely and sometimes we feel helpless against the disease, which cannot be compared to the worst sentimental deception that we may have had. Our humility is put to the test, and we are faced with our limits 1,2.

We also learn to value our time and to address the difficulties that we have in practicing the profession; we learn how difficult the task of giving bad news is, we make mistakes, and we lose our first patient. We find that sometimes we cannot offer the state-of-the-art treatment because it is not available in the public health system, especially in developing countries, but we can always do our best.

The health equipment in a university context is designed to train students and turn them into good doctors who will be responsible for taking care of the next generations. In this scenario, it is necessary to highlight the important role played by active agents in the propagation of knowledge, namely, our professors, masters and tutors.

The interpersonal relationship between students and trained doctors can take several forms, namely, medical visits with a didactic purpose, scientific orientation, lectures or even tutoring. Regardless of the form of interaction, it is imperative that the academic knowledge and personal experiences of each physician be passed on to younger generations, where they will find fertile, virgin ground to take root, develop and bear new fruits (whether scientific, human, social, etc.) for later generations.

I repeat, no matter how important and indispensable to the student medical books and fictitious clinical cases may be, they are insufficient to fully convey the biological, human, and social conceptions of the process of sickness and recovery. Throughout history, the medical student has never played an exclusively passive role in relation to the patient and the medicine 3.

An example worthy of note is the biography of Augusta Déjerine-Klumpke (1859-1927), an exponent of neuroscience. A brief glimpse into the history of her life is enough to prove her activism for women's rights in medicine, an exclusively male profession in Paris during her time. Her struggle for social inclusion echoes to this day, with more and more students discussing issues such as ethnoracial quotas for students in medical schools, for example.

Like Dr. Déjerine-Klumpke, students are the ones who break most paradigms. She was known not only for her activism in favor of women in the medical profession but also for her remarkable her prominence in the field of neuroanatomy throughout her academic life, which culminated in the description of lesions of the lower brachial plexus, later called "Paralysis of Klumpke".

A lot can be be said about many other students who have been involved in important scientific discoveries for medicine, which is certainly the case for Dr. Thomas J. Fogarty and the invention of the catheter bearing his name, for Dr. Auguste-Maurice Raynaud and the description of the Raynaud phenomenon, an important manifestation of rheumatological diseases, and for Charles Herbert Best (1899-1978) and Frederick Grant Banting (1891-1941) and the discovery of insulin 3, to name just a few.

It is remarkable how some of the great technical-scientific advances of medicine have come about through the hands of the then less-experienced but very creative students. Drawing a parallel with the theory of the Nobel laureate Dr. Daniel Kahneman, these young people have much more medical practice related to System II than to System I 4, unlike hospital seniors, for whom everything is more automatic, more fast-paced and more efficient but less innovative.

Such individuals were not afraid to dare to take a step toward the unknown, to defy valid ideas, and even to make mistakes. This same innovative spirit remains alive within students' hearts and can be stimulated and directed according to the action of experienced physicians and professors.

Therefore, yes, perhaps the health system exists "in spite of" the students, but it is their exposure to different environments, in essence, that hold the major responsibility for the training of a physician as a whole and, in turn, for the naive but attentive and creative look of the students that leads to the rupture of paradigms to achieve the more humane approach and perhaps to develop great scientific innovations. Let us not be afraid to participate actively, students!

Professors, we expect to see you in the next case discussions, whether in the basic health unit or in the ICU.[Fig f1-cln_73p1]

## Figures and Tables

**Figure f1-cln_73p1:**
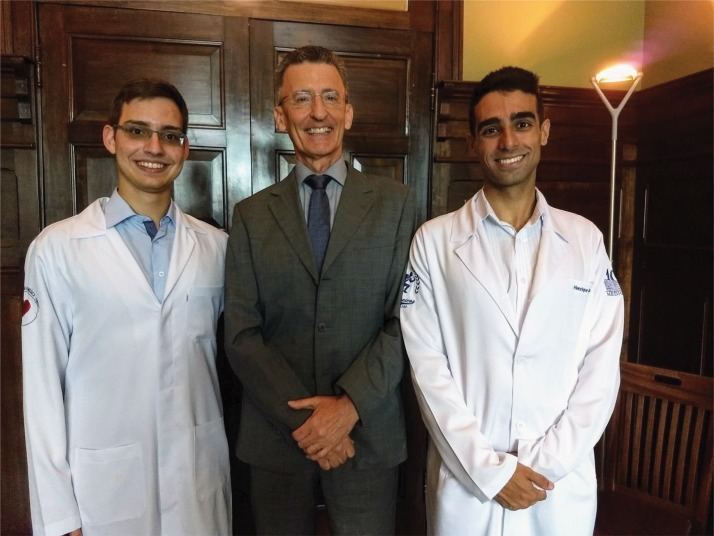
Gustavo Rosa Gameiro, Professor José Otavio Costa Auler Jr and Henrique Branzani da Silva ready for the next visit.

## References

[1] Bradley L (2017). Non-maleficence: perspective of a medical student. Br J Gen Pract.

[2] Ganguli I (2010). The case for primary care — a medical student’s perspective. N Engl J Med.

[3] Stringer MD, Ahmadi O (2009). Famous discoveries by medical students. ANZ J Surg.

[4] Kahneman D (2015). Thinking, fast and slow.

